# The intestinal microbiota regulates host cholesterol homeostasis

**DOI:** 10.1186/s12915-019-0715-8

**Published:** 2019-11-27

**Authors:** Tiphaine Le Roy, Emelyne Lécuyer, Benoit Chassaing, Moez Rhimi, Marie Lhomme, Samira Boudebbouze, Farid Ichou, Júlia Haro Barceló, Thierry Huby, Maryse Guerin, Philippe Giral, Emmanuelle Maguin, Nathalie Kapel, Philippe Gérard, Karine Clément, Philippe Lesnik

**Affiliations:** 1 INSERM, UMRS 1166, team “Integrative Biology of Atherosclerosis”, Sorbonne Universités, Hôpital Pitié-Salpêtrière, Paris, France; 2 Institute of Cardiometabolism and Nutrition (ICAN), Hôpital Pitié-Salpêtrière, Paris, France; 3 Neuroscience Institute and Institute for Biomedical Sciences, Georgia State University, Atlanta, GA USA; 4 INSERM, U1016, team “Mucosal microbiota in chronic inflammatory diseases”, Paris, France; 5 Institut Micalis, INRA, AgroParisTech, Université Paris-Saclay, Jouy-en-Josas, France; 6 Laboratoire de Coprologie Fonctionnelle, Hôpital Pitié-Salpêtrière, Paris, France; 7 EA 4065 “Ecosystème intestinal, probiotiques, antibiotiques”, Faculté des Sciences Pharmaceutiques et Biologiques Paris Descartes, Paris, France; 8 Sorbonne/INSERM, UMRS 1269, Nutrition et obésités : approches systémiques (nutriOmics), Hôpital Pitié-Salpêtrière, Paris, France; 9 Université de Paris, Paris, France

**Keywords:** Intestinal microbiota, Microbiome, Microbiota transfer, Cholesterol metabolism, Enterohepatic cycle, Antibiotics, Dyslipidemia

## Abstract

**Background:**

Management of blood cholesterol is a major focus of efforts to prevent cardiovascular diseases. The objective of this study was to investigate how the gut microbiota affects host cholesterol homeostasis at the organism scale.

**Results:**

We depleted the intestinal microbiota of hypercholesterolemic female *Apoe*^*−/−*^ mice using broad-spectrum antibiotics. Measurement of plasma cholesterol levels as well as cholesterol synthesis and fluxes by complementary approaches showed that the intestinal microbiota strongly regulates plasma cholesterol level, hepatic cholesterol synthesis, and enterohepatic circulation. Moreover, transplant of the microbiota from humans harboring elevated plasma cholesterol levels to recipient mice induced a phenotype of high plasma cholesterol levels in association with a low hepatic cholesterol synthesis and high intestinal absorption pattern. Recipient mice phenotypes correlated with several specific bacterial phylotypes affiliated to *Betaproteobacteria*, *Alistipes*, *Bacteroides*, and *Barnesiella* taxa.

**Conclusions:**

These results indicate that the intestinal microbiota determines the circulating cholesterol level and may thus represent a novel therapeutic target in the management of dyslipidemia and cardiovascular diseases.

## Background

Cholesterol is an essential component of eukaryotic cell membranes and is also a precursor of bile acids and steroid hormones. Dysregulation of cholesterol metabolism has been implicated in numerous diseases, including atherosclerosis and cardiovascular diseases [1], neurodegenerative diseases, non-alcoholic hepatitis [2], and cancers [3, 4]. Cholesterol metabolism is thus tightly regulated, and complex mechanisms regulate cholesterol levels, synthesis, and trafficking.

It has long been recognized that genetic [5, 6] and environmental factors such as the composition of the diet [7] as well as the amount of dietary cholesterol intake [8] have a strong impact on circulating cholesterol levels. Nevertheless, epidemiological studies recently reported that several bacterial taxa are associated with plasma cholesterol levels [9–11]. Other studies found a positive correlation between total and low-density lipoprotein (LDL) cholesterol and the abundance in the intestine of uncharacterized microbiota members belonging to *Erysipelotrichaceae* and *Lachnospiraceae* families [12]. Elevated plasma cholesterol and especially LDL cholesterol levels remain a major risk factor in cardiovascular diseases (CVD) [13–15]. While the contributing role of intestinal microbiota to CVD through the production of TMAO, a proatherogenic metabolite derived from dietary carnitine and phospholipids has been thoroughly demonstrated [16–19], recent data proposed that intestinal microbiota also impacts CVD pathogenesis through the modulation of circulating cholesterol levels. Moreover, dietary interventions showed that an increase in microbiota richness and diversity is associated with a decrease in circulating cholesterol [20, 21].

In normolipidemic wild type mice, germ-free (GF) condition as well as microbiota depletion through the administration of antibiotics upregulates de novo cholesterol synthesis with no raise in plasma cholesterol [22–24]. Until recently, the role of the intestinal microbiota in dyslipidemic experimental models that display a plasma lipoprotein profile closer to human, such as *Apoe*^*−/−*^ mice, has been poorly explored. Some publications have used pre- and probiotics to delineate how they can downregulates plasma cholesterol levels [25, 26]. The latest findings report that cholesterol levels and atherosclerosis lesions are higher in *Apoe*^*−/−*^ GF mice in comparison to *Apoe*^*−/−*^ conventionally raised mice but the mechanisms are still missing [27, 28]. Although transfer of intestinal microbiota into GF animals demonstrated that microbiota composition/activity determines recipient phenotype and susceptibility to several diseases [29, 30], this approach has not yet been applied for plasma cholesterol levels nor with human microbiota.

The objective of this study was to investigate how the gut microbiota affects host cholesterol homeostasis at the organism scale in a dyslipidemic context. First, we investigated how depleting the microbiota using antibiotics affects host cholesterol metabolism and cholesterol enterohepatic cycle. Then, using a strategy based on human to mice gut microbiota transplant, we demonstrate that specific intestinal microbiota composition regulates cholesterol absorption, biosynthesis, and circulating cholesterol levels.

## Methods

### Animal experimentation

*Apoe*^*−/−*^ and *LDLr*^−/−^ mice on the C57BL/6 J background were bred and kept in a conventional animal facility at the Central Animal Facility of La Pitié Salpêtrière Hospital, with temperatures maintained at 21 °C and with 12-h light and darkness cycles. Mice had free access to water and regular chow diet (RM1, Dietex) and were weaned at 22–24 days. All mice were anesthetized with isoflurane and then sacrificed by exsanguination and cervical dislocation.

### Intestinal microbiota depletion

Microbiota depletion was performed immediately after weaning by daily gavage with a combination of neomycin (200 mg/kg), metronidazole (200 mg/kg), ampicillin (200 mg/kg), and vancomycin (100 mg/kg) for 4 weeks [31]. Antibiotics were dissolved in tap water (20 mg/ml for neomycin, metronidazole, and ampicillin, and 10 mg/ml for vancomycin), filtered on 0.22 μm, aliquoted, and stored at − 20 °C until use. Control mice received water by oral gavage. All antibiotics were obtained from Sigma Aldrich.

### Gallbladder cannulation

Mice were anesthetized by an intraperitoneal injection of a mixture of ketamin and xylazine (100 mg/kg and 1 mg/kg, respectively). Mice were placed on a heating pad at 37 ± 1 °C. A 1.5-cm incision on the abdomen was performed, and the common bile duct was ligated. Then, a polyethylene tubing (0.023 in. diameter) was inserted in the gallbladder and maintained with another ligation. Bile was collected during 1 h in a 0.5-ml tube. The bile volume was assessed by pipetting.

### Intestinal microbiota transplant

Fresh human stool samples were collected in an anaerobic box (GENbag Anaert; Biomérieux). After thorough homogenization with a spatula, 1 g of stool was diluted (1:10 w/vol) and homogenized in reduced sterile Ringer solution (VWR) containing 0.5 g/L L-Cysteine (Sigma). This solution was then diluted 1:2 in reduced sterile 20% skimmed milk (Merk) and stored at − 80 °C until use. Mice were treated with antibiotics as previously described for 4 weeks in order to deplete their gut microbiota. After 2 h of fasting, mice were given 500 mg of polyethylene glycol (Colopeg, Bayer) by oral gavage to flush out antibiotics of their gut [32]. Six hours later, stool samples were thawed at 37 °C and mice were inoculated with 300 μl of the mixture. Then, mice were allowed free access to food. To ensure good colonization, mice were re-inoculated three additional times at days 1, 3, and 7. Mice were sacrificed and tissue collected 10 weeks later.

### Plasma lipids and lipoprotein profile

Total cholesterol, phospholipids, and triglycerides were analyzed with an autoanalyzer (Konelab) using commercial reagents from Roche Diagnostics and Diasys.

The lipid distribution in plasma lipoprotein fractions was assessed on pooled sera (*n* = 6 per group) by gel filtration as previously described [33]. Each fraction was subsequently analyzed for total cholesterol content as above.

### Intestinal cholesterol and bile acid absorption

To assess cholesterol absorption, mice were fasted overnight and then gavaged with 50 μCi [^3^H]-cholesterol dissolved in 250 μl of olive oil. Two hours later, the plasma and liver were collected. Ten microliters of plasma and 10 mg of liver were assayed for radioactivity in triplicates.

To assess bile acid absorption, mice were fasted overnight and then gavaged with 25 μCi [^3^H]-taurocholic acid dissolved in 250 μl of olive oil. Two hours later, the plasma and liver were collected. Ten microliters of plasma and 10 mg of liver were assayed for radioactivity in triplicates.

### Bile acid synthesis

Mice were gavaged with 50 μCi [^14^C]-cholesterol dissolved in olive oil. Then, feces were collected every 24 h during 72 h. Feces were dried at 60 °C during 1 h and manually grinded with a mortar. Two hundred milligrams of feces were homogenized in 1.2 ml of NaOH 0.5% in water and 1.2 ml of cyclohexane using a vortex during 2 min. Organic and aqueous phases were separated by centrifugation at 1200*g* during 10 min, collected, dried, and reconstituted in 200 μl of isopropanol and water, respectively. Ten microliters of each extract was assayed for radioactivity in triplicates.

### Gene expression analysis by quantitative PCR

Liver, ileum, or jejunum samples were disrupted in RNA-PLUS solution (QBiogene) using lysing matrix D in 2-ml tubes (MP Biomedicals) and Precellys homogenizer (Bertin technologies). Total RNA was extracted using Macherey-Nagel RNA extraction kit. RNA concentration and purity were determined using the Nanodrop ND-1000 spectrophotometer (Thermo Fisher Scientific) at a wavelength of 260/280 nm.

Total RNA (1.5 μg per reaction) was reverse transcribed into complimentary DNA using SuperScript II Reverse Transcriptase (Invitrogen) according to the manufacturer’s instructions. PCR amplification was performed in duplicates on cDNA diluted 1/100 using SYBR Green I Master and a Roche Lightcycler 480. The relative gene expression was calculated by the 2^-ΔΔCt^ calculation method, using 18S and hPRT as housekeeping genes and control group as reference.

### Sterols quantification in the liver and bile

Bile and liver lipids were extracted in the presence of two internal standards, pregnanol and 5α-cholestan (Steraloids), according to Folch et al. methodology [34]. Samples were homogenized in chloroform–methanol (2:1 v/v) using lysing matrix D in 2-ml tubes (MP Biomedicals) and a Precellys homogenizer (Bertin technologies). The organic extract was dried and reconstituted in methanol. Lipids were then saponified using 15% KOH (Sigma) in methanol at 60 °C during 1 h. Then, lipids were again extracted using hexan-diethyl-ether (1:1 v/v). The organic extract was subsequently dried and reconstituted in 60 μl cyclohexane, and silylation of sterols was performed with 40 μl of *N*,*O*-bis (trimethylsilyl)trifluoroacetamide-trimethylchlorosilane (99:1) at 60 °C during 1 h. Cholesterol and lathosterol were then quantified by GC-MS using a 5972 Hewlett Packard mass spectrometer and ChemStation data acquisition system. Briefly, sterols were injected in splitless mode and separated on a RTX65 column 30 m × 0.25 mm × 0.25 μm. Sterols were ionized using electronic impact and quantified in SIM mode. Ions 458.4 *m*/*z* and 255.0 *m*/*z* were used to quantify cholesterol and lathosterol, respectively.

### 16S rRNA gene sequencing

Feces were collected 3 to 5 days before sacrifice and immediately frozen in liquid nitrogen and then stored at − 80 °C. Fecal DNA was extracted as previously described [35]. The V3-V4 region of the 16S rRNA gene was amplified with the universal primers F343 (CTTTCCCTACACGACGCTCTTCCGATCTACGGRAGGCAGCAG) and R784 (GGAGTTCAGACGTGTGCTCTTCCGATCTTACCAGGGTATCTAATCCT), using 30 amplification cycles with an annealing temperature of 65 °C. The resulting PCR products were purified and sequenced at the GeT-PlaGe Genotoul INRA platform (Toulouse, France) using Illumina MiSeq technology. Sequences were trimmed for adaptors and PCR primer removal and then clustered into ASV using QIIME2. We picked a reference sequence for each ASV using Deblur and assigned it at different taxonomic levels (from phylum to species) using the Greengenes database 13_8 [36]. We used 99% sequence identity for ASV determination. The average number of sequences per sample was 5722 ± 1143 sequences per sample. Then, we normalized the dataset to the number of sequences of the sample with the lowest sequencing depth, that is to say 3619 sequences using Rhea script without random subsampling [37]. No sample was excluded from the downstream analyses as all the samples had a similar rarefaction curve terminal slope.

### Statistical analyses

Results are represented as mean ± SEM. Statistical analysis was performed by Mann–Whitney–Wilcoxon test using StatView Graphpad 6 (SAS Institute Inc., Cary, USA) for comparing two groups or by Kruskal–Wallis test followed by Dunn’s pairwise multiple comparisons procedure using R 3.3.1 program for comparing three or four groups; *p* or *q* < 0.05 was considered statistically significant. Principal component analyses (PCA) were performed using R program and ade4 package. Interclass PCA were computed and statistically assessed by a Monte Carlo rank test to observe their net effect on the scattering of the microbiota of different mice. We used R 3.3.1 and the Hmisc and corrplot packages to produce Spearman correlations matrix and the Rhea scripts pipeline to perform statistical analysis of the microbiota data [37]. The cladogram generator GraPhIAn was used for 16S data visualization [38].

## Results

### Microbiota depletion of conventional mice raises plasma cholesterol level

**Fig. 1:**
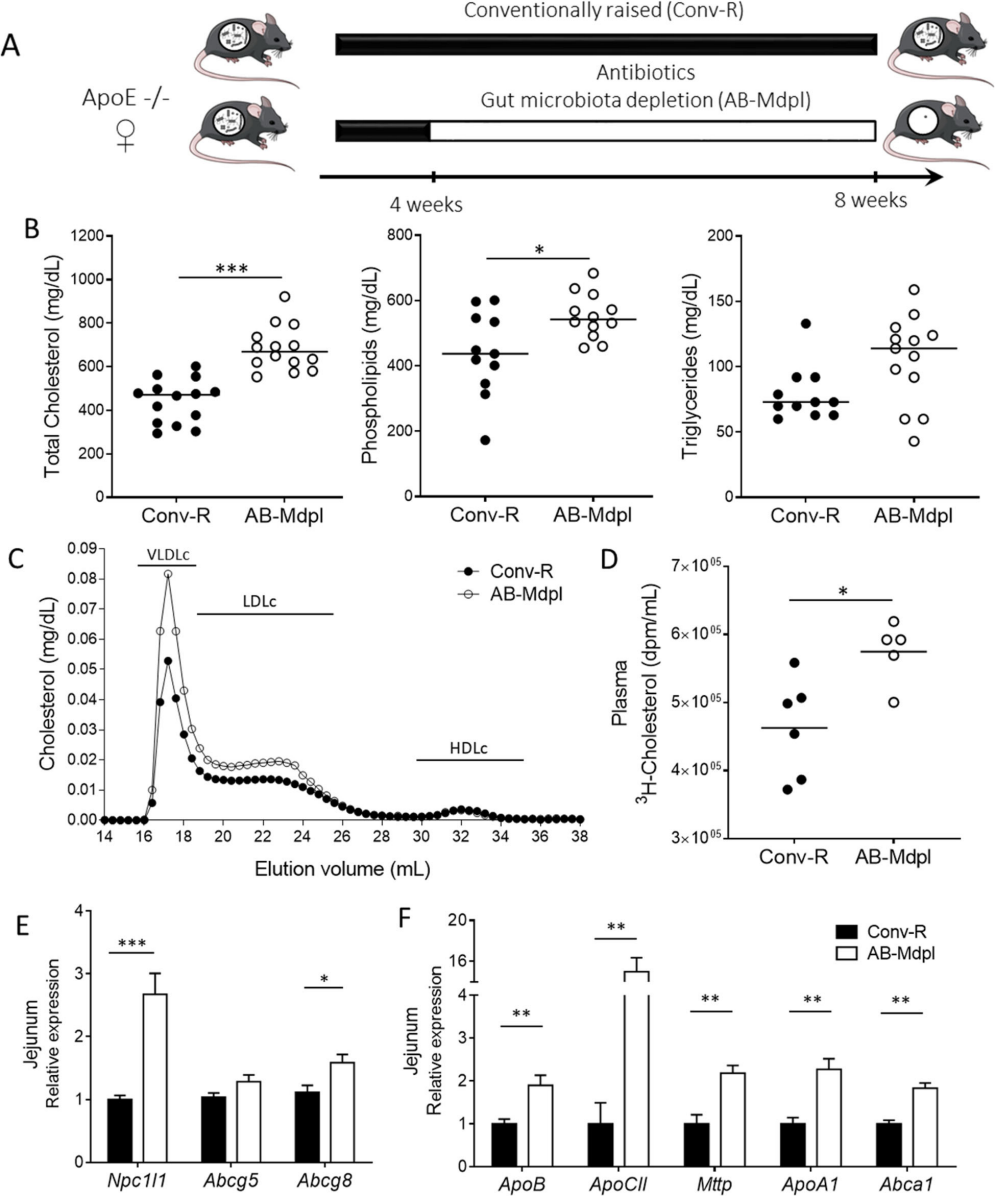
Intestinal microbiota depletion raises plasma cholesterol levels and intestinal cholesterol absorption. **a** Experimental design. See also Additional file 2: Figure S1. **b** Plasma cholesterol, phospholipids and triglycerides levels in conventionally raised (Conv-R) and microbiota-depleted mice (AB-Mdpl). **c** Cholesterol distribution across the VLDL, LDL, and HDL lipoprotein classes analyzed by fast protein liquid chromatography. **d** Plasma radioactivity 2 h after gavage with [^3^H]-cholesterol. **e** Relative expression of genes related to cholesterol absorption in the jejunum. **f** Relative expression of genes related to lipoprotein secretion in the jejunum. Data are represented as mean ± SEM, *n* = 5–10 mice/group (**d**, **e**) or as dots with median (**b**–**f**). Data were analyzed with Mann–Whitney test. *p < 0.05, **p < 0.01, ***p < 0.001

We aimed to decipher the role played by the intestinal microbiota in the regulation of plasma cholesterol levels in mice. To address this question, we depleted the gut microbiota of spontaneously hypercholesterolemic *Apoe*^*−/−*^ mice over 4 weeks by daily gavage with a mixture of antibiotics consisting of vancomycin, ampicillin, neomycin, and metronidazole (Fig. 1a, Additional file 1). After 7 days of treatment, intestinal microbiota depletion was effective and stable during 3 weeks with a copy number of 16S rRNA genes in feces 10^5^-fold less than the initial bacterial load (Additional file 2: Figure S1) in agreement with previous findings [31]. Plasma total cholesterol level was 55% higher in microbiota-depleted (AB-Mdpl) mice compared with conventionally raised (Conv-R) mice (Fig. 1b). Plasma phospholipids and triglycerides were also raised by microbiota depletion, although not statistically significant for triglycerides (Fig. 1b).

Cholesterol in the plasma exists mainly packaged in the form of lipoproteins: chylomicrons, very-low-density lipoproteins (VLDL), low-density lipoproteins (LDL), and high-density lipoproteins (HDL). Quantitative analysis of cholesterol distribution among lipoproteins separated by gel filtration revealed an increase of the abundance of VLDL and LDL subclasses (respectively + 53% and + 36%) in AB-Mdpl mice, whereas the HDL fraction was similar in Conv-R and AB-Mdpl mice (Fig. 1c).

These experiments confirm that intestinal microbiota contribute to the regulation of plasma cholesterol levels and demonstrate that microbial depletion strongly affect several lipoproteins levels, mainly VLDL and LDL.

### Intestinal microbiota depletion increases intestinal cholesterol absorption with no effect on hepatic VLDL production

As the liver secretes VLDL particles, we investigated the impact of microbiota depletion on VLDL production. Likewise, as LDL particles derive from the loss of triglycerides by VLDL and intestine originating chylomicrons in the bloodstream, we investigated intestinal cholesterol absorption.

Then, we measured the appearance of labeled cholesterol in the plasma of conventionally raised and microbiota-depleted mice 2 h following gavage of [^3^H]-cholesterol in olive oil. The appearance of radiolabeled cholesterol in the plasma was 25% higher in antibiotic-treated mice (Fig. 1d), indicating that depleting the microbiota raises intestinal cholesterol absorption.

We next analyzed the jejunal expression of genes involved in intestinal cholesterol absorption (*Npc1l1* [39]) and intracellular cholesterol excretion in the gut lumen (*Abcg5* and *8* [40]). We observed that microbiota-depleted mice displayed a threefold increase in *Npc1l1* expression while *Abcg8* expression was moderately raised and *Abcg5* expression was not affected (Fig. 1e). Moreover, the expression of several genes encoding apolipoproteins and proteins involved in chylomicron and preβ-HDL assembly and secretion were increased at least two folds in the jejunum of microbiota-depleted mice (Fig. 1f).

VLDL are assembled in the liver from triglycerides, cholesterol, and apolipoproteins (ApoB mainly) by the chaperone Mttp. Here, liver gene expression levels of *ApoB* and *Mttp* of Conv-R and AB-Mdpl mice were similar (Additional file 3: Figure S2A). This is consistent with the similar VLDL secretion rate assessed using Triton WR-1339 as an inhibitor of peripheric lipid uptake by endothelial lipoprotein lipase [41] (Additional file 3: Figure S2B).

This set of experiments reveals that depleting the intestinal microbiota with antibiotics raises intestinal cholesterol absorption. On the contrary, the hypothesis of elevated VLDL levels in microbiota-depleted mice being a consequence of increased hepatic VLDL synthesis and secretion is rather unlikely.

### Intestinal microbiota depletion increases the hepatic clearance of plasma cholesterol through LDLr

**Fig. 2:**
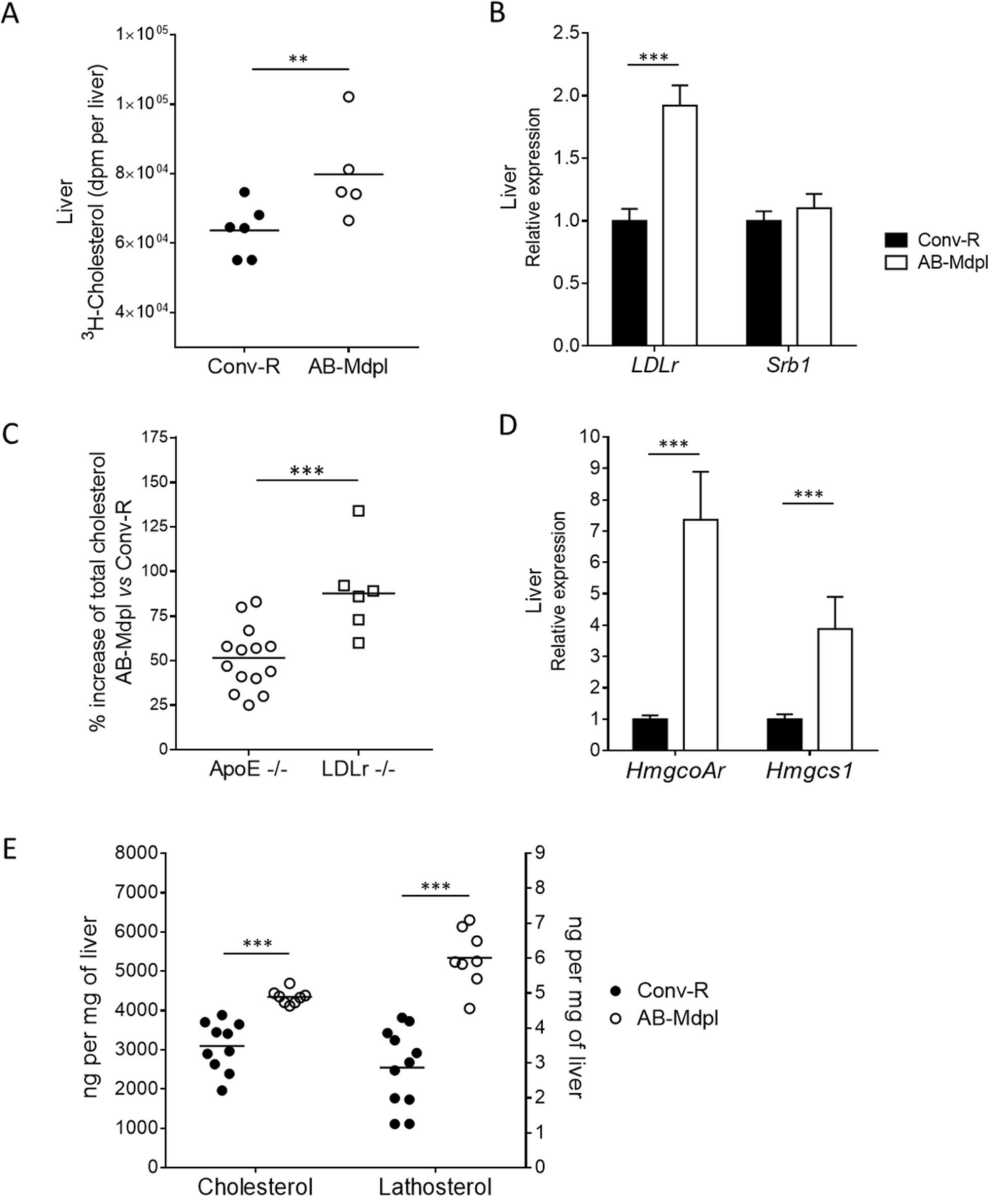
Intestinal microbiota depletion increases hepatic cholesterol uptake and hepatic cholesterol synthesis. **a** Liver radioactivity 2 h after gavage with [^3^H]-cholesterol in conventionally raised (Conv-R) and microbiota-depleted mice (AB-Mdpl). **b** Hepatic relative expression of cholesterol transporters. **c** Plasma cholesterol increase in microbiota-depleted mice in comparison to control mice in Apoe (○) and LDLr (□)^−/−^ mice. **d** Hepatic relative expression of genes related to cholesterol synthesis. See also Additional file 5: Figure S3. **e** Cholesterol and lathosterol concentration analyzed by GC-MS in the liver. Data are represented as mean ± SEM, *n* = 6–9 mice/group (**b**–**d**) or as dots with median (**a**, **c**, **e**). Data were analyzed with Mann–Whitney test. *p < 0.05, **p < 0.01, ***p < 0.001

[^3^H]-cholesterol absorption assay demonstrated that the level of radiolabeled cholesterol was 37% higher in the liver of microbiota-depleted mice (Fig. 2a, Additional file 4), suggesting a microbial regulation of hepatic cholesterol uptake. The uptake of cholesterol-rich particles HDL and LDL into the liver is mediated by their respective receptors, scavenger receptor type B1 (SR-B1) and LDL receptor (LDLr) [42]. mRNA levels of *LDLr* were significantly increased by microbiota depletion which was not the case for *SR-B1* mRNA (Fig. 2b). Hence, we submitted *LDLr*^*−/−*^ mice to the same microbiota depletion protocol and measured their circulating cholesterol levels. Strikingly, microbiota depletion raised plasma cholesterol levels by 91% in LDLr-deficient mice against only 50% in Apoe-deficient mice (Fig. 2c). This demonstrates that LDLr-mediated cholesterol uptake by the liver partially counteracts the plasma cholesterol raise induced by microbiota depletion.

### Intestinal microbiota depletion enhances cholesterol synthesis in the liver

The gastrointestinal tract contributes to 15–35% and the liver to 20–40% of total cholesterol synthesis in rodents [43]. The relative expression of *Hmgcs1* and *HmgcoAr*, encoding two key enzymes in cholesterol biosynthesis pathway, was not affected following intestinal microbiota depletion in the intestine (Additional file 5: Figure S3) but significantly increased by four- to sevenfold in the liver (Fig. 2d). We next determined the liver content of cholesterol and lathosterol, a synthesis intermediate considered as a marker of cholesterol synthesis [44], by gas chromatography coupled to mass spectrometry (GC-MS). Cholesterol concentration was 30% higher and lathosterol concentration was doubled in the liver of AB-Mdpl compared to Conv-R mice (Fig. 2e). This indicates that intestinal microbiota regulates cholesterol biosynthesis specifically in the liver.

### The intestinal microbiota influences bile acid synthesis and biliary cholesterol secretion

**Fig. 3:**
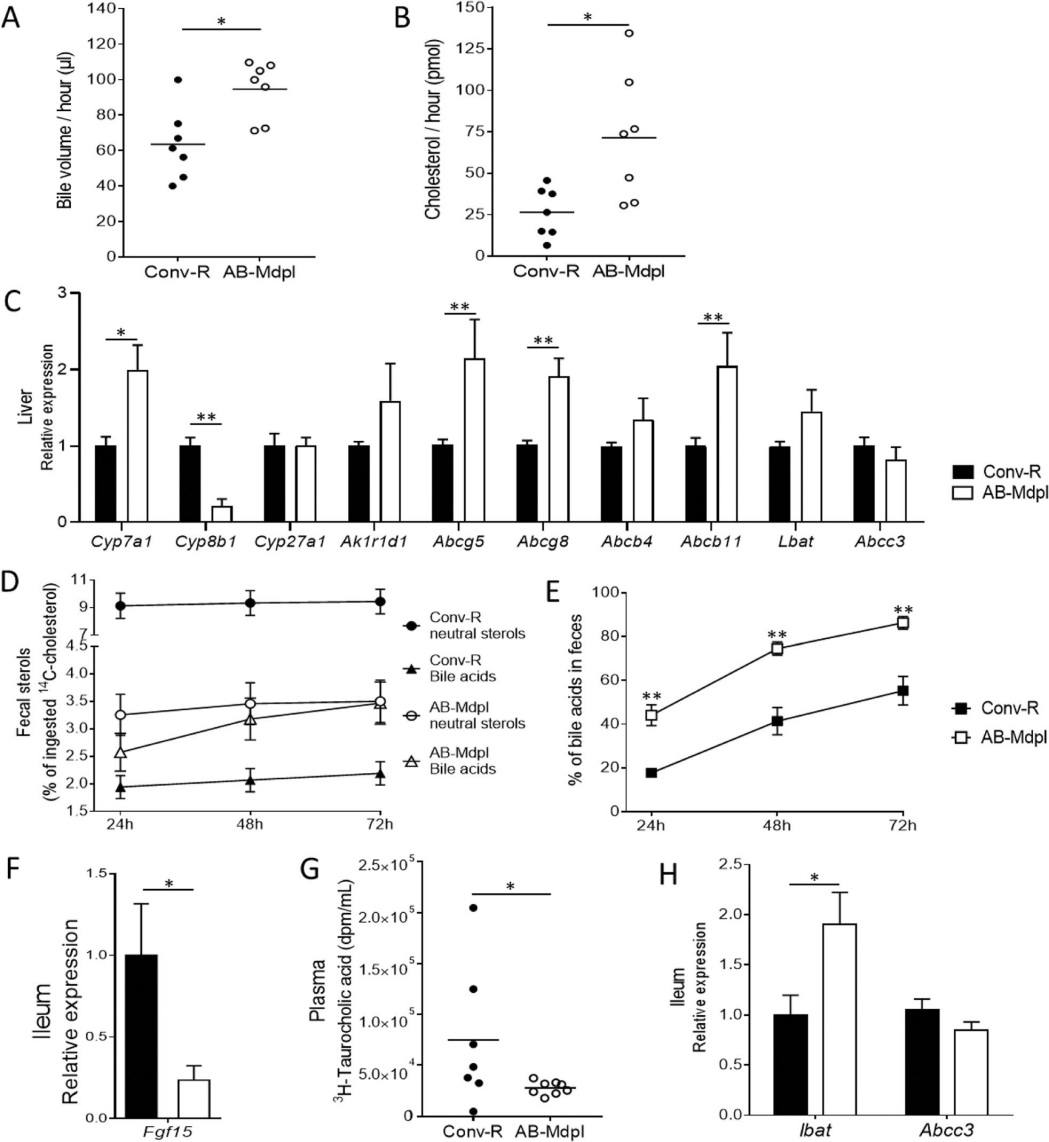
Enterohepatic cycle of cholesterol and bile acids in conventionally raised and microbiota-depleted mice. **a** Bile volume collected in 1 h of gallbladder cannulation in conventionally raised (Conv-R) and microbiota-depleted mice (AB-Mdpl). **b** Quantity of cholesterol secreted in the bile during 1 h of gallbladder cannulation. **c** Hepatic gene expression of enzymes involved in bile acid biosynthesis and of transporters of cholesterol and bile acids in conventionally raised (Conv-R) and microbiota-depleted mice (AB-Mdpl). **d** Fecal excretion of ^14^C bile acids (water-soluble fraction) and ^14^C cholesterol (cyclohexane soluble fraction) during 72 h after oral gavage with ^14^C cholesterol. **e** ^14^C bile acids excreted in the feces expressed as percent of total radioactivity (cholesterol + bile acids). **f** Relative expression of *fgf15* in the distal ileum. **g** Plasma radioactivity 2 h after gavage with [^3^H]-taurocholic acid. **h** Relative gene expression of bile acid transporters in the distal ileum. Data are represented as mean ± SEM (**c**, **f**, **h**) or as dots with median (**a**, **b**, **g**), *n* = 5–8 mice/group. Data were analyzed with Mann–Whitney test. *p < 0.05, **p < 0.01, ***p < 0.001

Cholesterol is mainly excreted from the body in the bile that is then secreted in the duodenum, leading to fecal excretion in two forms: cholesterol and bile acids. To evaluate cholesterol output from the liver, we monitored bile flow during 1 h and found a 40% increase in AB-Mdpl mice compared to control mice (Fig. 3a**,** Additional file 6). We demonstrated that biliary cholesterol secretion in the intestinal lumen was significantly increased in AB-Mdpl mice compared to controls (Fig. 3b). Importantly, cholesterol is apically secreted from hepatocytes to bile as free cholesterol via ABCG5/8 [45], whose gene expression was twofold greater in AB-Mdpl mice (Fig. 3c).

The drastic depletion of intestinal microbiota increases intraluminal cholesterol absorption as well as re-excretion in the bile by the liver. To investigate how intestinal microbiota depletion influences the balance between cholesterol intake and secretion, we force-fed mice with ^14^C-cholesterol and collected their feces every 24 h during 72 h. We separated neutral lipids containing cholesterol from water-soluble components including bile acids and measured radioactivity in each fraction (Fig. 3d). Conv-R mice excreted 70% more radioactive sterols (sum of neutral lipids and water-soluble fraction) than AB-Mdpl over 72 h (Fig. 3d), confirming that sterols accumulated in the body in the absence of microbiota. Specifically, AB-Mdpl mice excreted threefold less cholesterol and 50% more bile acids than Conv-R mice; hence, the bile acids represented a significantly higher proportion of fecal sterols in Ab-Mdpl mice (Fig. 3e). This suggests that the absence of gut microbiota leads to an accumulation of sterols in the body and that in this context bile acids constitute a significant proportion of fecal sterols.

Next, we observed that the increased fecal bile acid excretion was associated with a regulation of enzymes in the bile synthesis pathway. Expression levels of *Ak1r1d1* and *Cyp7a1*, the rate-limiting enzyme in the bile acid synthesis pathway, were increased in the liver in AB-Mdpl mice, supporting an increased bile acid synthesis in the absence of microbiota (Fig. 3c). However, *Cyp27a1* expression was similar in both groups while *Cyp8b1* expression was decreased in AB-Mdpl mice (Fig. 3c). Considering that microbiota is known to induce intestinal FXR which in turn regulates hepatic Cyp7a1 through a fibroblast growth factor 15 (Fgf-15)-dependent mechanism [46], we determined *Fgf-15* expression in the distal ileum. We found that microbiota depletion reduces *Fgf-15* expression by 75% (Fig. 3f).

As microbiota depletion raises bile acid synthesis and secretion, we needed to examine whether modification of intestinal bile acid absorption can strengthen or lessen fecal loss of bile acids. Gavage with ^3^H-taurocholic acid showed that microbiota depletion significantly decreases taurocholic acid absorption (Fig. 3g). This is probably not related to a decrease in active transport of bile acids, as the gene expression of the two transporters Ibat and Abcc3 was not decreased by the microbiota depletion (Fig. 3h). This decrease in taurocholic acid absorption is therefore likely the consequence of a decrease in passive absorption, the major absorption pathway of microbiota-derived unconjugated bile acids [47].

### Plasma cholesterol level is transmissible from humans to mice by microbiota transplantation

**Fig. 4:**
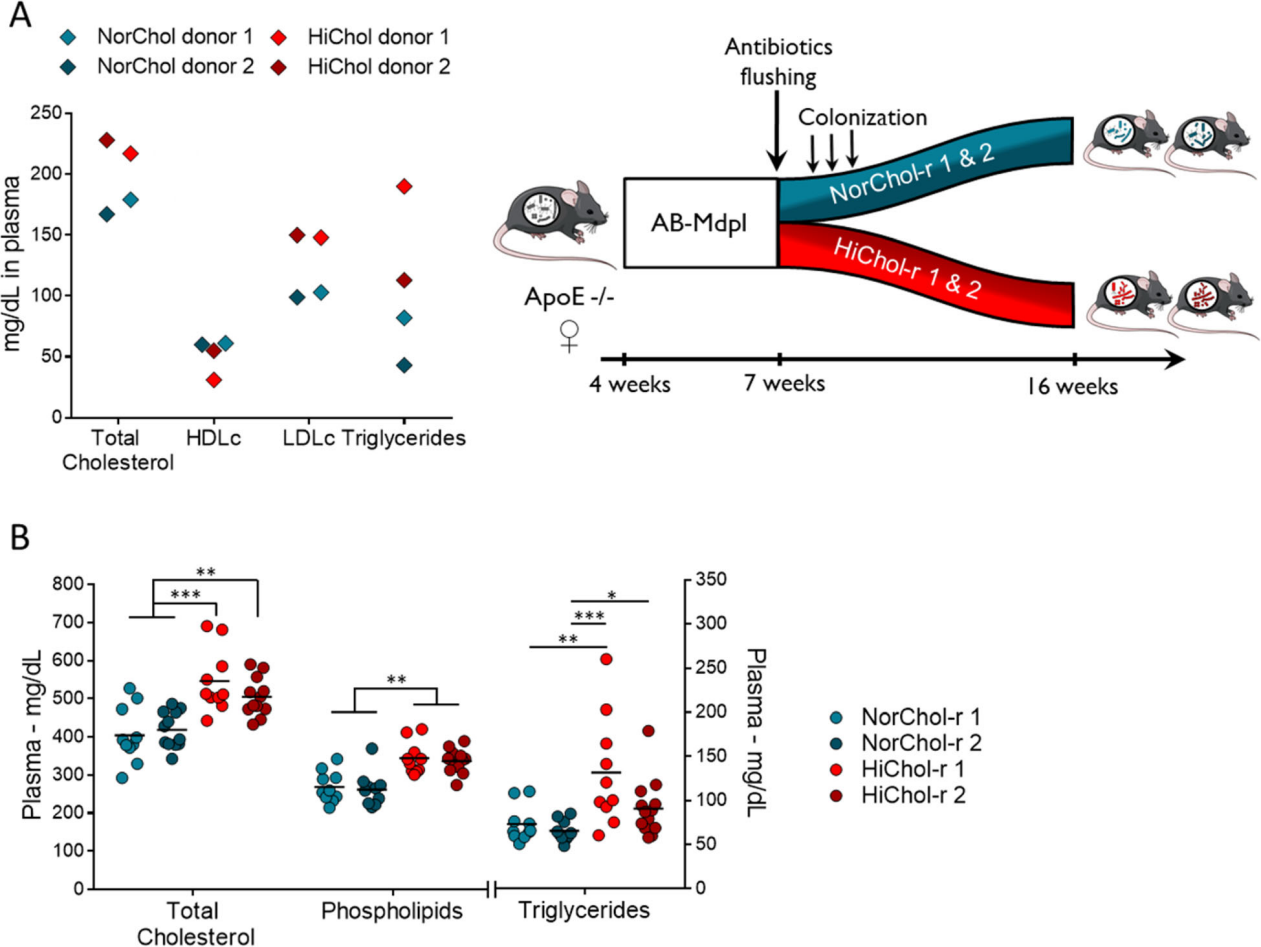
Plasma cholesterol levels are transferable from humans to mice by intestinal microbiota transplantation. **a** Donors’ characteristics and experimental design. **b** Plasma cholesterol, phospholipids, and triglycerides levels in mice colonized with the microbiota from normocholesterolemic donors (NorChol-r1 and r2, pictured cyan and dark cyan) and high-cholesterol donors (HiChol-r1 and r2, pictured in red and dark red). Data are represented as dots with median (**a**, **b**), *n* = 8–12 mice/group. Recipient groups were analyzed using Kruskal–Wallis test followed by Dunn’s pairwise multiple comparison procedure. *q < 0.05, **q < 0.01, ***q < 0.001

Our first experiments indicated that the lack of a functional microbiota deeply disrupts host cholesterol metabolism. We therefore hypothesized that not only bacterial load will impact cholesterol metabolism, but also that variations in intestinal microbiota composition and functionality might induce variations of cholesterol circulating levels. We thus selected human microbiota donors whose plasma cholesterol levels were discrepant and colonized recipient mice with their intestinal microbiota. We selected four women based on their plasma lipid profile: two donors with normal blood cholesterol levels (NorChol) and two donors with moderately elevated total cholesterol levels (HiChol) (Fig. 4a, Additional file 7). These subjects received no treatment. Consistently with a dyslipidemic context, HDL cholesterol levels were slightly lower in the two HiChol donors while LDL cholesterol and triglycerides levels were considerably higher (Fig. 4a).

We colonized four groups of microbiota-depleted 7-week-old female *Apoe*^*−/−*^ mice (*n* = 10–14 mice per group) through repeated oral gavages with fecal microbiota from respective donors (Fig. 4a). Strikingly, after 9 weeks, the mean of plasma total cholesterol levels of HiChol recipient mice was 23% higher than those of NorChol recipients (Fig. 4b). Other plasma lipids such as triglycerides and phospholipids were also dramatically increased (Fig. 4c), suggesting that as their donors, HiChol recipient mice had an overall altered plasma lipid profile.

### Intestinal microbiota regulates cholesterol absorption/synthesis balance

**Fig. 5:**
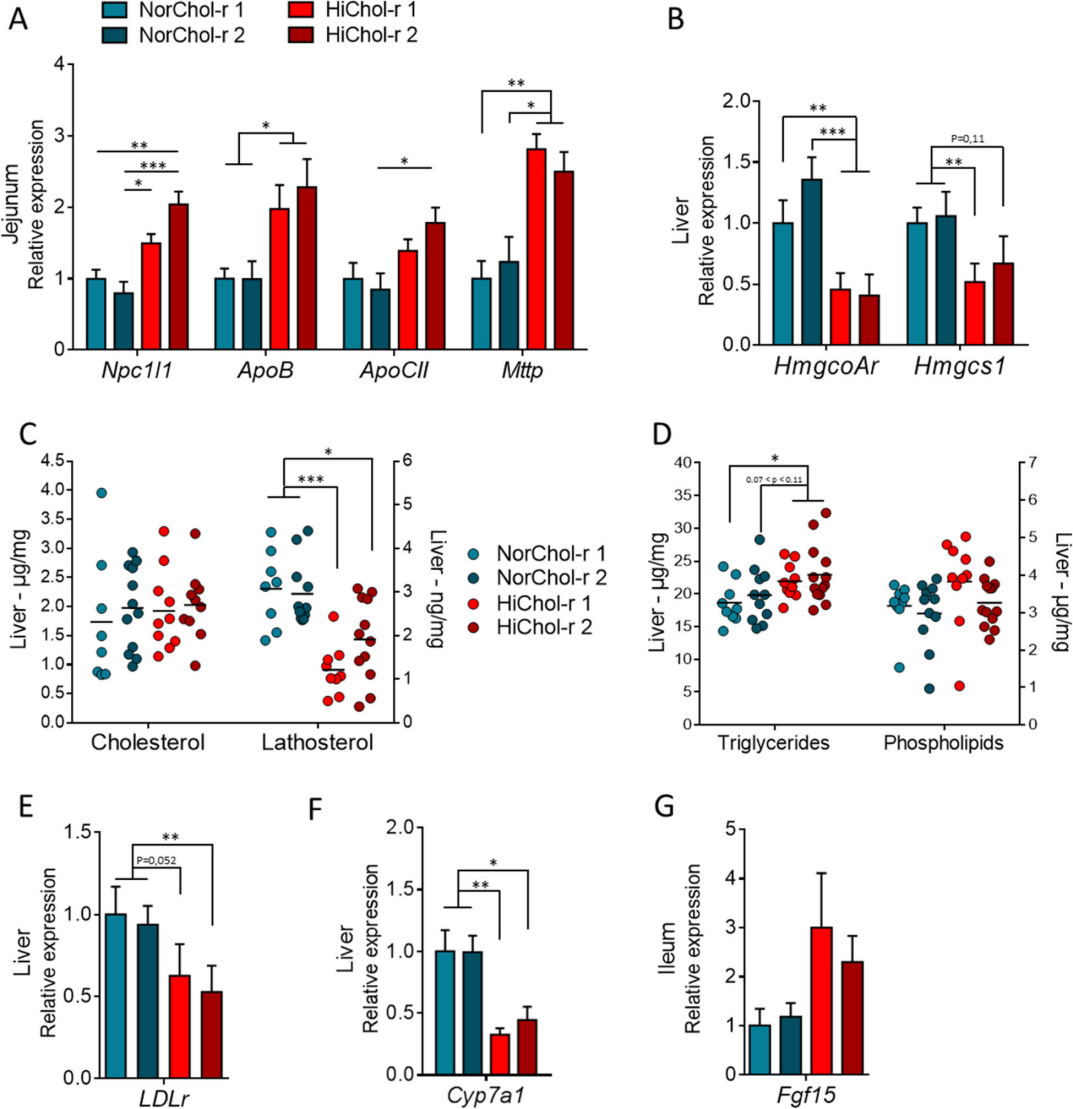
Intestinal microbiota regulates cholesterol absorption/synthesis balance. **a** Relative expression of genes related to cholesterol absorption and lipoprotein secretion in the jejunum in mice colonized with the microbiota from normocholesterolemic donors (NorChol-r1 and r2, pictured cyan and dark cyan) and high-cholesterol donors (HiChol-r1 and r2, pictured in red and dark red). **b** Relative expression of enzymes involved in cholesterol synthesis in the liver. See also Additional file 9: Figure S4. **c** Cholesterol and lathosterol concentration analyzed by GC-MS in the liver. **d** Triglycerides and phospholipids analyzed by biochemic assay in the liver. **e** Hepatic relative expression of *LDLr*. **f** Hepatic relative expression of *Cyp7a1* in the liver. **g** Relative expression of *fgf15* in the distal ileum. Data are represented as mean ± SEM (**a**, **b**, **e**, **f**, **g**) or as dots with median (**c**, **d**), *n* = 8–12 mice/group. Recipient groups were analyzed using Kruskal–Wallis test followed by Dunn’s pairwise multiple comparison procedure. *q < 0.05, **q < 0.01, ***q < 0.001

To investigate if intestinal microbiota from dyslipidemic or normolipidemic donors could modulate cholesterol metabolism pathways, we analyzed the expression in the jejunum of genes related to intestinal cholesterol absorption and lipoprotein secretion. *Npc1l1*, *ApoB*, *ApoCII*, and *Mtpp* were all significantly more expressed in both HiChol recipient groups than in both NorChol recipient groups (Fig. 5a, Additional file 8). This suggests that the intestinal microbiota from dyslipidemic donors upregulates intestinal cholesterol absorption in recipient mice compared to mice colonized with microbiota from normolipidemic donors.

On the contrary, genes of the cholesterol synthesis pathway (*HmgcoAr* and *Hmgcs1*) were two times less expressed in the liver of HiChol recipients than in Norchol recipients (Fig. 5b). Consistently, the concentration of lathosterol was significantly lower in the liver of the two groups of HiChol recipients than in the liver of Norchol recipients, supporting a decrease in hepatic cholesterol synthesis in HiChol recipient mice (Fig. 5c). However, hepatic cholesterol content was not affected by the donors’ status (Fig. 5c), suggesting that other cholesterol metabolism pathways in the liver were affected by the microbiota. As cholesterol, liver phospholipids were similar in the four groups while liver triglycerides were slightly raised in HiChol recipients in comparison to NorChol recipients (Fig. 5d).

Hepatic expression of LDL receptor was lower in HiChol than in NorChol recipient mice (Fig. 5e), suggesting a decreased hepatic uptake in mice colonized with the microbiota from dyslipidemic donors. Moreover, the expression of *Cyp7a1* was also reduced in HiChol recipients, which likely result from the increased expression of its suppressor *Fgf15* in the distal ileum (Fig. 5f, g). There was a trend towards decreased *Cyp8b1* and canalicular cholesterol *Abcg5/g8* and bile acid *Abcb11* transporters, but this did not reach statistical significance (Additional file 9: Figure S4).

Altogether, this set of experiments suggests an elevated intestinal cholesterol absorption and a decreased hepatic uptake and synthesis in HiChol recipient mice in comparison to NorChol recipient mice. Biliary cholesterol secretion in the intestinal lumen may also be lower in HiChol than in NorChol recipient mice. This indicates more broadly that the microbiota could be a regulator of the intestinal absorption/hepatic synthesis balance.

### Mice colonized with the microbiota of normocholesterolemic and dyslipidemic human donors harbor distinct intestinal microbiota composition

**Fig. 6:**
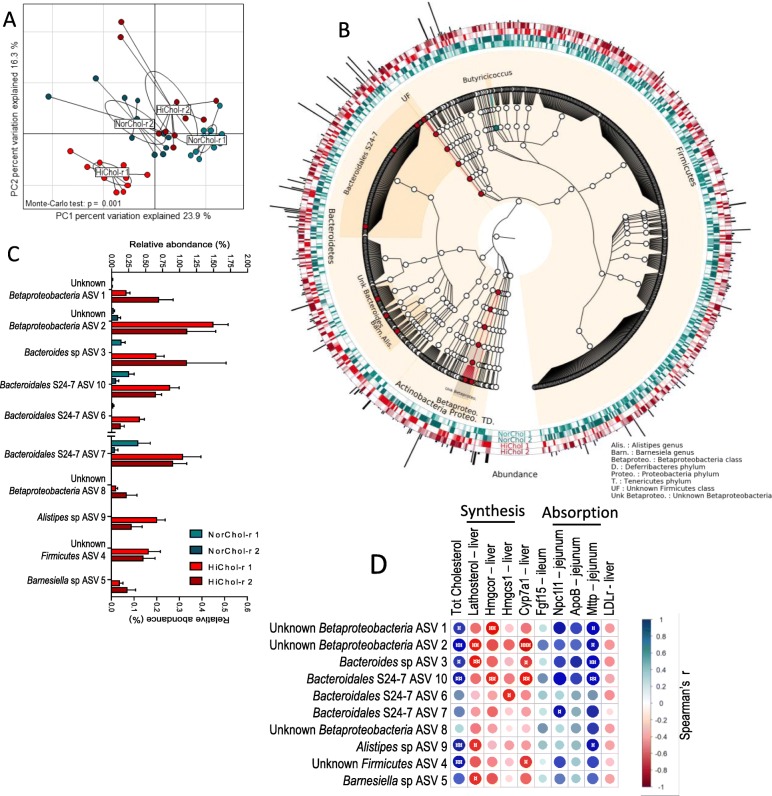
Mice colonized by the microbiota of normocholesterolemic and high-cholesterol human donors harbor specific intestinal microbiota composition. **a** Interclass principal component analysis performed based on ASVsabundance. Mice microbiota were clustered and the center of gravity computed for each group. The *p* value of the link between recipient groups and ASV abundance was calculated using a Monte Carlo test (999 replicates). **b** Cladogram generated using GraPhlAn [38] representing recipients’ microbiota with cyan clade-markers highlighting bacterial groups significantly more abundant in NorChol recipients and red clade-markers highlighting bacterial groups significantly more abundant in HiChol recipients as assessed by Kruskal–Wallis test followed by Dunn’s pairwise multiple comparison procedure. Circular heatmap represents normalized abundance of all ASV in each recipient group, with the darkest color corresponding to the group having the highest percentage of the given ASV. Black bars represent the mean abundance of the ASVs in the whole data set. **c** Bacterial ASVs statistically more abundant in both HiChol recipients’ groups than in both NorChol recipients’ groups. *n* = 9–12 mice/group. **d** Spearman correlations between ASV-level microbial populations and cholesterol metabolism-associated parameters. Strong correlations are indicated by large circles, whereas weaker correlations are indicated by small circles. The colors of the circles denote the nature of the correlation with dark blue indicating strong positive correlation and dark red indicating a strong negative correlation. ^**¤**^*q* < 0.05, ^**¤¤**^*q* < 0.01, ^**¤¤¤**^*q* < 0.001 after FDR correction

In order to identify bacterial species or taxa involved in the regulation of cholesterol homeostasis, we analyzed by 16S rRNA gene sequencing of the V3-V4 region the fecal microbiota of recipient mice 9 weeks after colonization. Richness, Simpson, and Shannon alpha diversity indices were similar between recipient mice groups (Additional file 10: Figure S5). Interclass PCA based on the ASV abundance showed that the microbiota of mice clustered separately depending on the microbiota donor (Fig. 6a). The two NorChol and the two HiChol recipient groups did not cluster together. We then looked for ASVs that were specifically over- or underrepresented in both NorChol groups in comparison to both HiChol groups, and no particular phylum was differently represented in NorChol and HiChol recipient mice (Fig. 6b and Additional file 11: Figure S6). After assignation to lower taxonomic levels and cladogram construction using GraPhlAn [38], we found that *Betaproteobacteria* class was significantly more abundant in both HiChol recipient groups of mice than in both NorChol recipient mice groups (Fig. 6b and Additional file 11: Figure S6). This was mainly due to higher proportions of unclassified *Betaproteobacteria* (Fig. 6b and Additional file 12: Figure S7). Unclassified Firmicutes were also found in higher proportions in the microbiota of HiChol recipient mice (Fig. 6a, c, d, and Additional file 12: Figure S7 A and B). Ten ASVs corresponding to 6 taxonomic clusters were found to be more abundant in HiChol recipient’s microbiota (Fig. 6c). Three members of the Bacteroidales S24-7 class were more abundant in HiChol recipients than in NorChol recipients, as well as one ASV related to *Bacteroides* genus, one related to *Alistipes* genus and *Barnesiella* genus (Fig. 6c). In addition, 3 ASVs belonging to unclassified *Betaproteobacteria* and one to unclassified Firmicutes were specifically associated with HiChol recipients.

### HiChol-associated microbiota taxa correlate with plasma cholesterol levels

To confirm whether one or several specific gut bacteria were involved in the regulation of major cholesterol metabolism pathways, we performed multiple correlation analyses between the previously identified ASVs and plasma cholesterol level as well as parameters associated with hepatic cholesterol synthesis, lipoprotein uptake by the liver, bile acid synthesis, and intestinal absorption (Fig. 6d). Six of the ten HiChol recipient-associated ASVs were significantly and positively correlated with plasma cholesterol levels. Five of these ASVs correlated negatively with markers of hepatic cholesterol synthesis such as *HmgcoAr* expression and lathosterol concentration in the liver. These ASVs also positively correlated with markers of intestinal absorption such as *Npcl1* and *Mttp* expression in the jejunum. The *Fgf15* expression in the ileum and the *LDLr* expression in the liver were also correlated with these ASVs; however, statistical significance was not reached, suggesting that these parameters of cholesterol metabolism are less tightly regulated by the microbiota than the other parameters. The sequences of seven of these ten ASVs were not assigned to the genus level by Qiime2 pipeline; however, manual BLAST against the EzBioCloud 16S data base (update 06 august 2019) [48] indicated that ASV 1 belongs to the *Sutterellaceae* family, ASV 3 and ASV 8 belong to the *Turicimonas* genus, and ASV 4 to the *Erysipelotrichaceae* family.

## Discussion

**Fig. 7:**
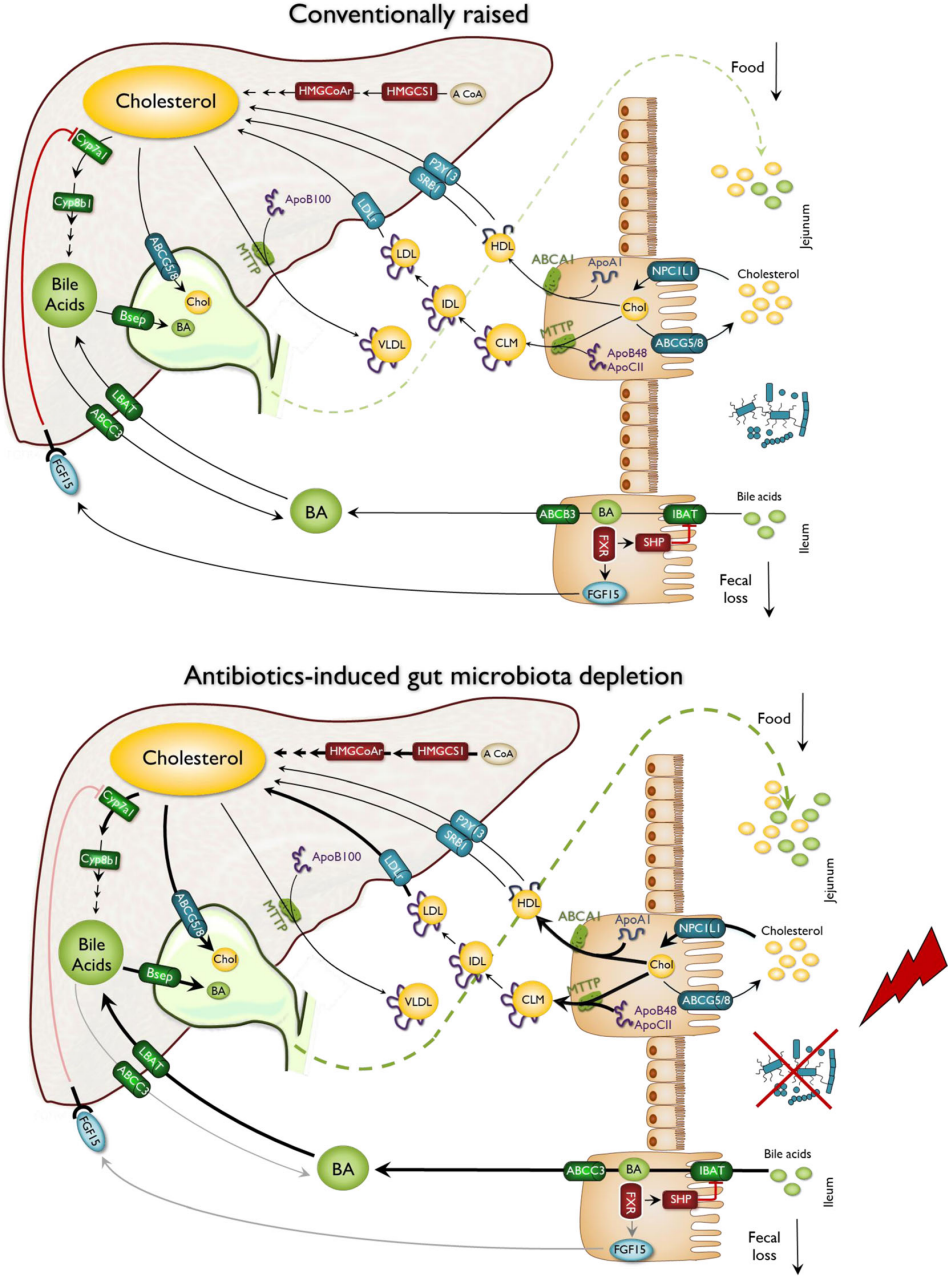
Microbial regulation of whole-body cholesterol fluxes and enterohepatic cycle. Microbiota depletion in *Apoe*^*−/−*^ mice raises plasma VLDL and LDL cholesterol. Microbiota-depleted mice have increased intestinal cholesterol absorption, hepatic cholesterol uptake, and hepatic cholesterol and bile acid synthesis. Bile secretion is also increased in microbiota-depleted mice, which is associated with increased fecal excretion of bile acids. Microbiota depletion is associated with a decrease in *fgf15* expression in the distal ileum, thus alleviating feedback inhibition of hepatic bile acid synthesis

Cholesterol is an essential lipid and component of eukaryotic cellular membrane and precursor for bile acids and steroid hormone synthesis. Its elevated concentration in the bloodstream is considered as a hallmark of cardiovascular diseases in humans. In the present study, we investigated the contribution of the gut microbiota in the regulation of plasma cholesterol levels and, more generally, to cholesterol homeostasis. We demonstrated that depleting the gut microbiota using antibiotics raises plasma cholesterol levels and profoundly alters cholesterol metabolism in Apoe-deficient mice. Indeed, depleting the intestinal microbiota increases intestinal cholesterol and bile acid absorption, lipoprotein secretion by the intestine, hepatic cholesterol uptake via LDL receptor, and hepatic cholesterol and bile acid synthesis as well as bile secretion in the intestinal lumen. This reflects an amplification of both cholesterol and bile acid enterohepatic cycles (Fig. 7). These functional experiments were substantiated at the molecular level since a deep depletion of the microbiota using antibiotics cocktail strongly altered the expression of key genes in the jejunum, ileum, and liver.

We found that the expression in the liver of genes involved in bile acid secretion was downregulated in the presence of a complete microbiota, in accord with previous studies [46]. We also observed a downregulation of cholesterol transporters *Abcg5/8*, following antibiotic exposure, consistent with previous studies in germ-free mice [24] but discrepant with others [22]. Nevertheless, our data on *Abcg5/8* expression are reinforced by the observation of a decrease in bile flow and cholesterol excretion in the presence of a complex microbiota. This dissimilarity could be the consequence of differences in diets and mice strains, with Rabot et al. and Ceasar et al. studies using wild type mice and high-fat diets with different fat composition and proportions, while in the present work we used dyslipidemic mice fed a chow, low-fat diet. Taken together, these observations support the growing idea that biological processes and in particular cholesterol metabolism can be determined by microbe–nutrient interactions and not only by microbiota and/or diet taken independently [22, 24, 49].

In our model, we also observed that the intestinal microbiota lowered intestinal absorption of dietary and biliary cholesterol, which are the following steps of enterohepatic circulation (Fig. 7). Intraluminal cholesterol is transported across the apical membrane of enterocytes by *Npc1l1* [50], which expression was downregulated by the presence of microbiota. This somewhat surprising finding is opposed to the previous study addressing the impact of microbiota on intestinal cholesterol absorption [51]. In both cases, *Npc1l1* expression in the small intestine could be related to the effective intestinal cholesterol absorption measured by radioactivity tracking techniques. *Npc1l1* transcriptional regulation is not so well described yet and existing data do not converge towards the same theory. However, mice were fed a western diet rich in lipids and sucrose in Zhong et al. study. *Npc1l1* intestinal expression may be modulated by glucose [52] and SREBP2 [53], which are greatly affected by western diet only in the presence of a microbiota. We speculate that, once again, nutrients regulate cholesterol homeostasis depending on the presence or absence of intestinal microbiota. Moreover, *Npc1l1* expression is decreased by a *Lactobacillus* strain [54] and prebiotic fibers [55]. This confirms the ability of specific bacteria to decrease intestinal cholesterol absorption through *Npc1l1* transcriptional downregulation in the intestine.

The following step of the enterohepatic circulation of cholesterol is the recapture of the absorbed cholesterol by the liver, where endogenous synthesis also occurs (Fig. 7). Indeed, the liver plays a pivotal role in cholesterol metabolism and plasma cholesterol levels as it (i) captures most of circulating cholesterol, (ii) secretes cholesterol-containing VLDL particles in the bloodstream, (iii) synthesizes significant amounts of cholesterol, and (iv) secretes cholesterol in the bile or converts it into bile acids (Fig. 7). We observed that both cholesterol uptake by the LDL receptor and hepatic de novo synthesis are drastically downregulated by the intestinal microbiota. We observed that the lack of microbiota triggered a downregulation of *Fgf15* expression, which in turn suppresses the downregulation of bile acid synthesis in the liver, which corroborates several studies [46, 51], but is discrepant with others [22]. The disruption of this *Fgf15*-dependent regulation pathway by microbiota depletion mainly explains the elevation of cholesterol synthesis and cholesterol uptake by the liver. The presence of intestinal microbiota is thus essential to whole-body cholesterol homeostasis, and we observed that the dysregulation of cholesterol enterohepatic cycle caused by microbiota depletion leads to an increase in cholesterol concentration in each compartment, notably in the plasma.

This raises the important question of the impact of variations of microbiota composition on the plasma cholesterol level in humans. To explore this, we colonized microbiota-depleted mice with the microbiota of four participants whose plasma lipid profile (total cholesterol, HDL/LDL cholesterol ratio, and triglycerides levels) was either healthy or associated with CVD risk according to the European and American cardiovascular societies [13, 56]. Murine recipients from dyslipidemic donors had significantly higher plasma cholesterol levels compared with recipients from normocholesterolemic donors. Thus, not only the presence/absence of intestinal microbiota, but also the variations in intestinal microbiota composition are sufficient to influence plasma cholesterol level. Each group of recipient mice had a specific microbial community, and very few features discriminate the cholesterol metabolism patterns. This confirms the high inter-variability in human microbiota composition and suggests that the majority of intestinal bacteria have no impact on host cholesterol metabolism while a limited number of taxa have a significant impact. Among the few bacteria that were associated with HiChol status, we found one ASV belonging to the *Erysipelotrichaceae* family, which has already been found to be positively correlated to plasma cholesterol [9, 12]. We were also able to positively correlate plasma cholesterol level and cholesterol metabolism to other bacterial taxa such as *Alistipes*, *Barnesiella*, and *Turicimonas*, which to our knowledge have not yet been associated with cholesterol metabolism. Notably, HiChol-associated bacteria were more strongly correlated with cholesterol metabolism-associated parameters than NorChol-associated bacteria. Hence, this suggests that the observed phenotypes in this study were likely the consequence of the presence of some deleterious bacteria rather than the absence of beneficial bacteria.

Several bacterial taxa are believed to lower plasma cholesterol levels or to reduce atherosclerosis development through the production of beneficial metabolites such as butyrate [57], through bile acid metabolism, or through entrapment of cholesterol [58]. Conversely, the deleterious effect of gut microbiota on atherosclerosis development via TMAO production has been studied [17], but no mechanism explaining how some bacteria can raise plasma cholesterol has been described. Our study highlight that some bacteria are probably able to exert such deleterious activity; however, the fact that most of those bacteria are not cultivable at the present time prevents the study of the involved mechanism.

Finally, an important observation made in this study is that mice colonized with the microbiota of dyslipidemic donors had markers of elevated intestinal cholesterol absorption together with a lower cholesterol synthesis, while mice colonized with the microbiota of normocholesterolemic donors exhibited an opposite pattern. It is of high clinical interest that intestinal microbiota is able to shift the cholesterol absorption/synthesis balance, since it has repeatedly been observed in human cohorts that high absorption/low synthesis pattern is associated with higher LDL cholesterol and lower HDL cholesterol level and is predictive of CVD events [44, 59–61]. Strikingly, those high CVD risk individuals are those who have the poorest response to statins, the most used cholesterol-lowering drugs which act by inhibiting cholesterol synthesis in the liver [62].

## Conclusions

In this study, we unveil the influence of intestinal microbiota on cholesterol fluxes and synthesis at the whole-body scale. By combining a series of in vivo investigations based on microbiota manipulation in dyslipidemic mice models, we report how the intestinal microbiota regulates cholesterol synthesis, absorption, and trafficking. Importantly, we show that plasma cholesterol levels can be transferred from humans to mice by intestinal microbiota transplantation, demonstrating the causal role of microbiota in the regulation of plasma cholesterol levels. We also show that intestinal microbiota regulates the balance between cholesterol synthesis and absorption. Thus, our findings open new possibilities for the prevention and treatment of CVD through modulation of the microbiota composition by the use of prebiotics, probiotics, or fecal transplantation.

## Supplementary information

**Additional file 1: Table S1.** Datapoints for Fig. 1.

**Additional file 2: Figure S1.** Antibiotic treatment efficiently depletes intestinal microbiota. Bacterial 16S DNA load in feces before and after 7 days of antibiotics treatment. 16S DNA was determined by quantitative PCR and the mean of the bacterial load before antibiotic treatment was normalized to 100%. Data were analyzed with Mann-Whitney test., ** *p* < 0.01.

**Additional file 3: Figure S2:** microbiota depletion does not alter hepatic VLDL production. (A) Hepatic relative expression of genes related to VLDL production in conventionally raised (Conv-R) and microbiota depleted mice (AB-Mdpl). (B) Triglycerides accumulation in the blood of tyloxapol injected mice, reflecting VLDL secretion. Data are represented as mean ± SEM, *n* = 8–10 mice / group. Data were analyzed with Mann-Whitney test.

**Additional file 4: Table S2.** Datapoints for Fig. 2.

**Additional file 5: Figure S3.** Antibiotics-induced microbiota depletion does not affect intestinal cholesterol synthesis. Jejunal and ileal expression of HmgcoA reductase in conventionally raised (Conv-R) and microbiota depleted mice (AB-Mdpl). Data are represented as mean ± SEM, n = 8–10 mice / group. Data were analyzed with Mann-Whitney test.

**Additional file 6: Table S3.** Datapoints for Fig. 3.

**Additional file 7: Table S4.** Datapoints for Fig. 4.

**Additional file 8: Table S5.** Datapoints for Fig. 5.

**Additional file 9: Figure S4.** Relative expression in the liver of normocholesterolemic and high cholesterol recipient mice. Relative expression of genes related bile acid synthesis (Cyp8b1) and bile secretion (Abcg5, 8 and 11) in the liver of mice colonized with the microbiota from normocholesterolemic donors (NorChol-r1 and r2, pictured cyan and dark cyan) and high cholesterol donors (HiChol-r1 and r2, pictured in red and dark red). Data are represented as mean ± SEM, *n* = 10–12 mice / group. Data were analyzed with Kruskal–Wallis test followed by Dunn’s pairwise multiple comparisons procedure.

**Additional file 10: Figure S5.** Mice colonized by the microbiota of normocholesterolemic and high cholesterol human donors harbor similar gut microbiota alpha-diversity. (A) Microbial richness in index in mice associated with human microbiota (NorChol-r1 and r2, pictured cyan and dark cyan, HiChol-r1 and r2, pictured in red and dark red). (B) Effective Simpson diversity index in mice associated to human microbiota. (C) Effective Shannon diversity index in mice associated with human microbiota. Data were analyzed with Kruskal–Wallis test followed by Dunn’s pairwise multiple comparisons procedure.

**Additional file 11: Figure S6.** Fecal microbiota composition of normocholesterolemic and high cholesterol recipient mice. (A) Bacterial phyla distribution as percentage of total sequences in mice colonized with the microbiota from normo-cholesterolemic and high-cholesterol donors. (B) Bacterial classes distribution as percentage of total sequences in recipient mice (NorChol-r1 and r2, pictured cyan and dark cyan, HiChol-r1 and r2, pictured in red and dark red). (C) Bacteria orders as percentage of total sequences in recipient mice. (D) 12 most abundant families as percentage of total sequences in recipient mice. (E) 12 less abundant families as percentage of total sequences in recipient mice. (E) Data are represented as box and whiskers (10–90 percentile), n = 10–12 mice / group. * *p* < 0.05, ** *p* < 0.01, *** *p* < 0.001.

**Additional file 12: Figure S7.** Fecal microbiota composition of normocholesterolemic and high cholesterol recipient mice. (A) 12 most abundant genera as percentage of total sequences in recipient mice (NorChol-r1 and r2, pictured cyan and dark cyan, HiChol-r1 and r2, pictured in red and dark red). (B) 12 less abundant genera as percentage of total sequences in recipient mice. (C) 12 low abundant genera as percentage of total sequences in recipient mice. Data are represented as box and whiskers (10–90 percentile), n = 10–12 mice / group. * p < 0.05, ** p < 0.01, *** p < 0.001.

## Data Availability

The raw data of 16S rRNA gene libraries generated during this study is publicly available at the Sequence Read Archive (SRA) portal of NCBI under accession number PRJNA543019 [63]. The other data generated or analyzed during this study are included in this published article and its supplementary information files.

## Abbreviations

AB-Mdpl: Antibiotic-induced microbiota depletion
Conv-R: Conventionally raised
CVD: Cardiovascular diseases
GC-MS: Gas chromatography–mass spectrometry
GF: Germ-free
PCA: Principal component analysis
HDL: High-density lipoproteins
LDL: Low-density lipoproteins
ASV: Amplicon sequence variant
VLDL: Very-low-density lipoproteins
