# Supplementary figures and images for: The intestinal microbiota regulates host cholesterol homeostasis
Source: BMC Biol. 2019 Nov 27;17:94. doi: 10.1186/s12915-019-0715-8 (PMC6882370; doi:10.1186/s12915-019-0715-8)

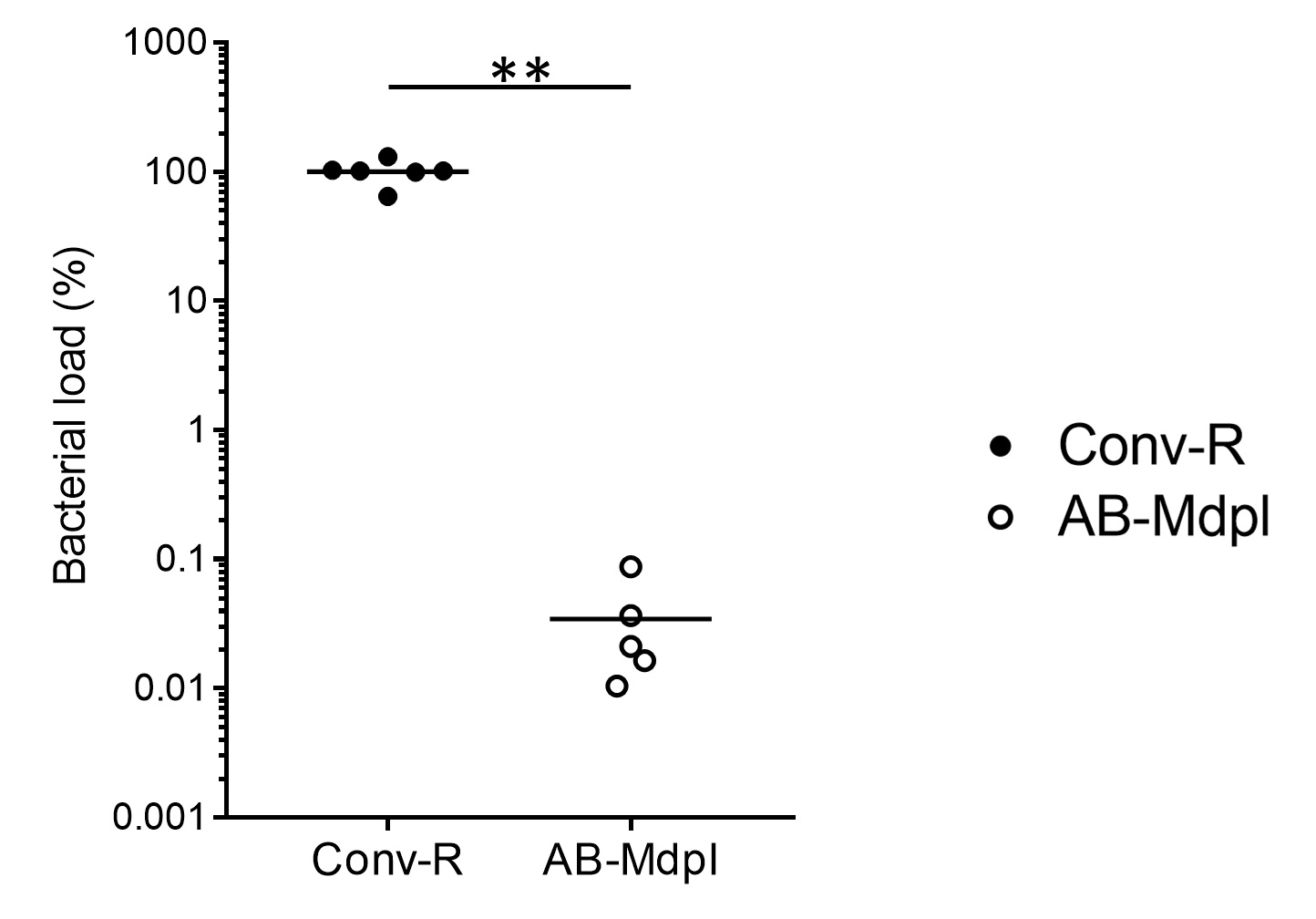

Supplement: Supplementary file 2 — Additional file 2: Figure S1. Antibiotic treatment efficiently depletes intestinal microbiota. Bacterial 16S DNA load in feces before and after 7 days of antibiotics treatment. 16S DNA was determined by quantitative PCR and the mean of the bacterial load before antibiotic treatment was normalized to 100%. Data were analyzed with Mann-Whitney test., ** p < 0.01. [file 12915_2019_715_MOESM2_ESM.jpg]

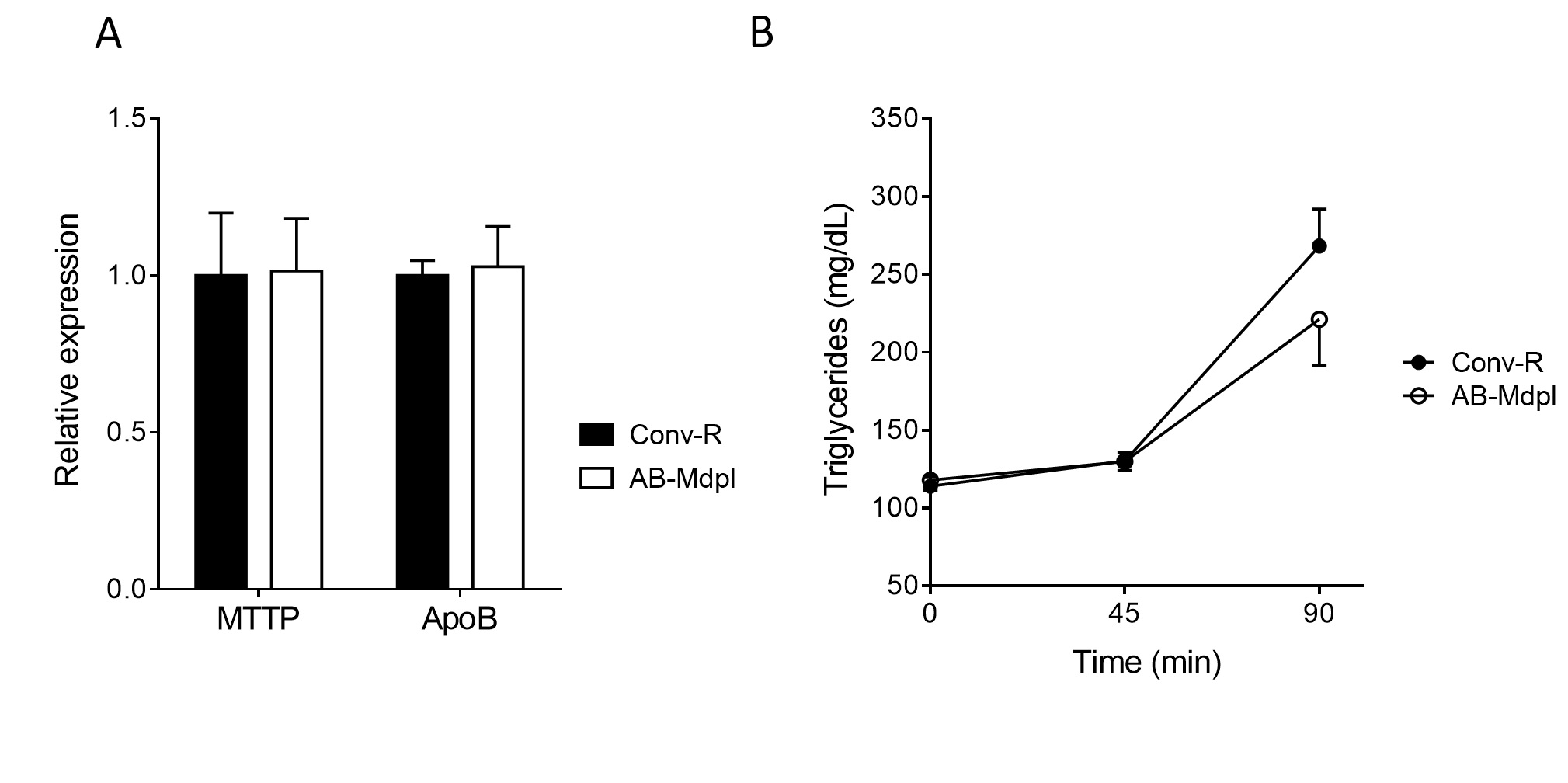

Supplement: Supplementary file 3 — Additional file 3: Figure S2: microbiota depletion does not alter hepatic VLDL production. (A) Hepatic relative expression of genes related to VLDL production in conventionally raised (Conv-R) and microbiota depleted mice (AB-Mdpl). (B) Triglycerides accumulation in the blood of tyloxapol injected mice, reflecting VLDL secretion. Data are represented as mean ± SEM, n = 8–10 mice / group. Data were analyzed with Mann-Whitney test. [file 12915_2019_715_MOESM3_ESM.jpg]

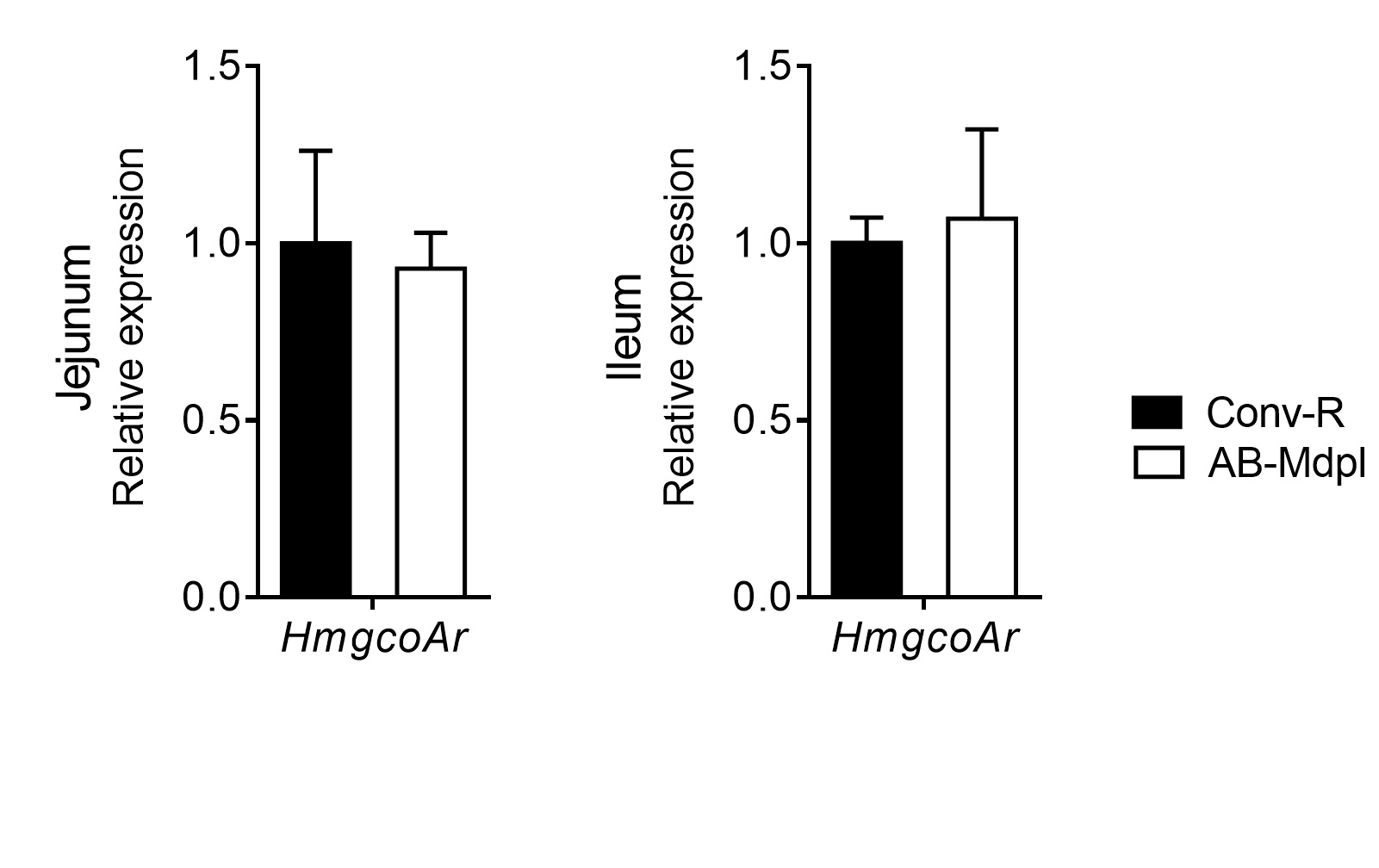

Supplement: Supplementary file 5 — Additional file 5: Figure S3. Antibiotics-induced microbiota depletion does not affect intestinal cholesterol synthesis. Jejunal and ileal expression of HmgcoA reductase in conventionally raised (Conv-R) and microbiota depleted mice (AB-Mdpl). Data are represented as mean ± SEM, n = 8–10 mice / group. Data were analyzed with Mann-Whitney test. [file 12915_2019_715_MOESM5_ESM.jpg]

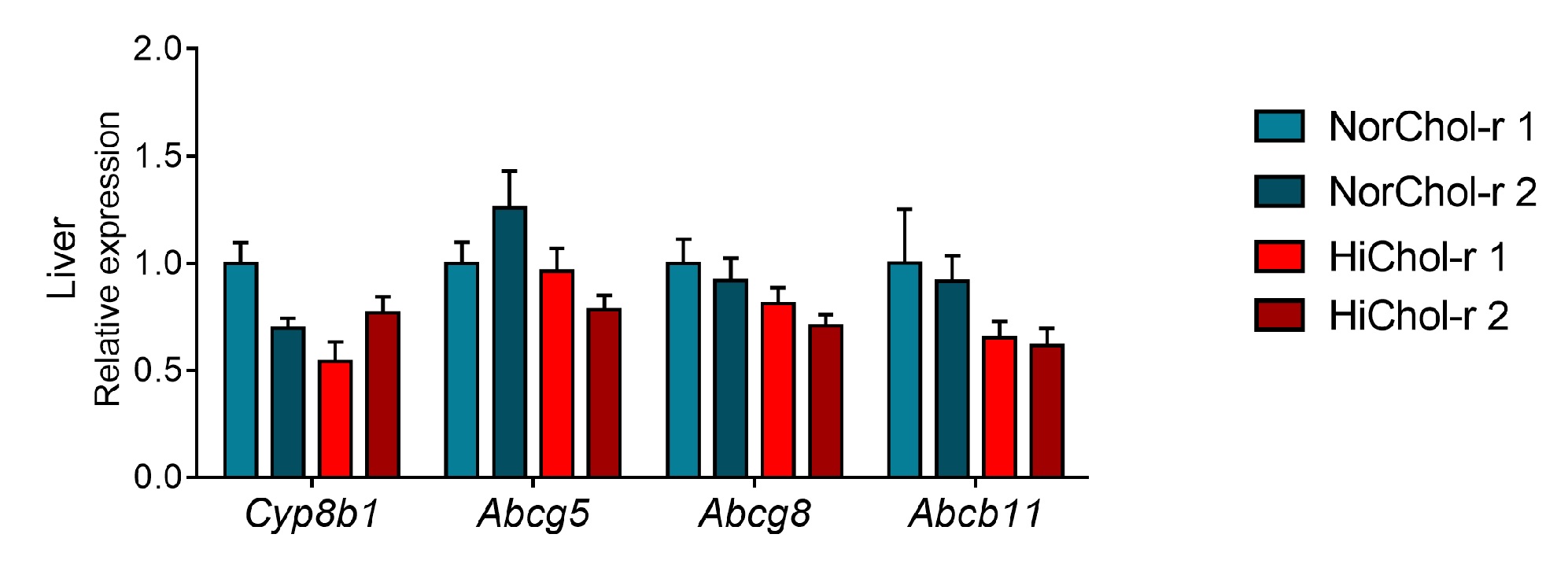

Supplement: Supplementary file 9 — Additional file 9: Figure S4. Relative expression in the liver of normocholesterolemic and high cholesterol recipient mice. Relative expression of genes related bile acid synthesis (Cyp8b1) and bile secretion (Abcg5, 8 and 11) in the liver of mice colonized with the microbiota from normocholesterolemic donors (NorChol-r1 and r2, pictured cyan and dark cyan) and high cholesterol donors (HiChol-r1 and r2, pictured in red and dark red). Data are represented as mean ± SEM, n = 10–12 mice / group. Data were analyzed with Kruskal–Wallis test followed by Dunn’s pairwise multiple comparisons procedure. [file 12915_2019_715_MOESM9_ESM.jpg]

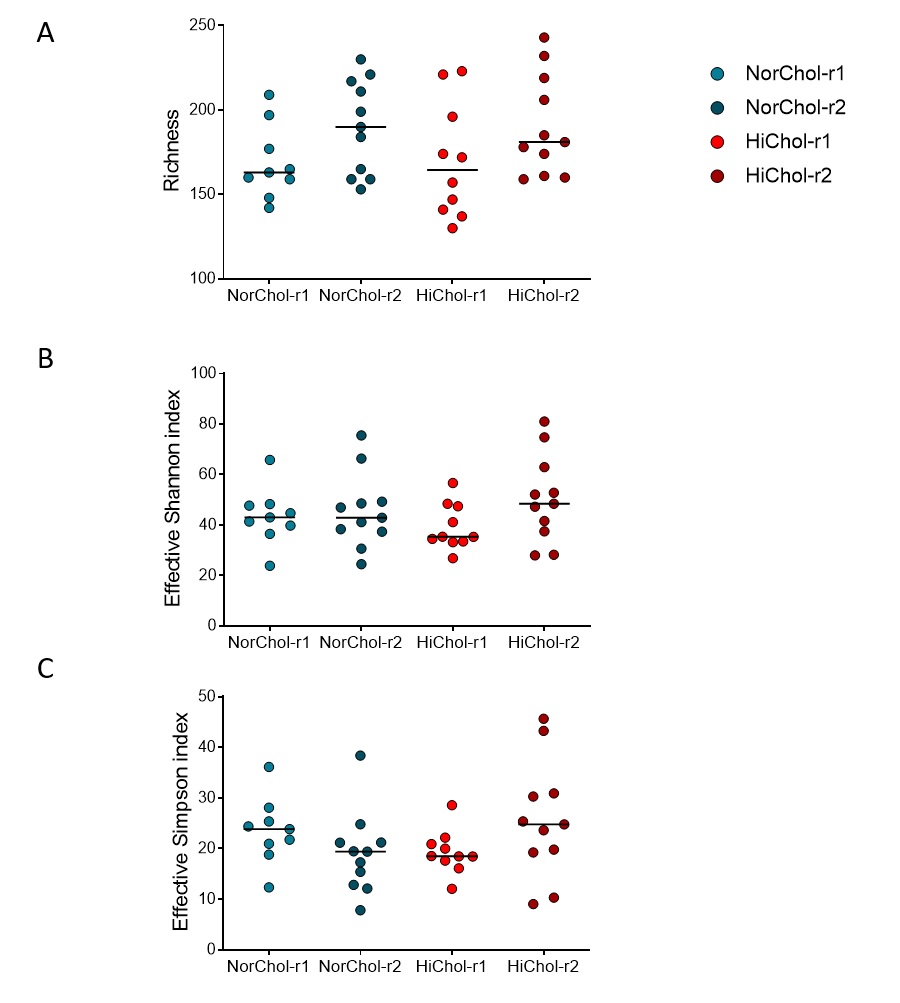

Supplement: Supplementary file 10 — Additional file 10: Figure S5. Mice colonized by the microbiota of normocholesterolemic and high cholesterol human donors harbor similar gut microbiota alpha-diversity. (A) Microbial richness in index in mice associated with human microbiota (NorChol-r1 and r2, pictured cyan and dark cyan, HiChol-r1 and r2, pictured in red and dark red). (B) Effective Simpson diversity index in mice associated to human microbiota. (C) Effective Shannon diversity index in mice associated with human microbiota. Data were analyzed with Kruskal–Wallis test followed by Dunn’s pairwise multiple comparisons procedure. [file 12915_2019_715_MOESM10_ESM.jpg]

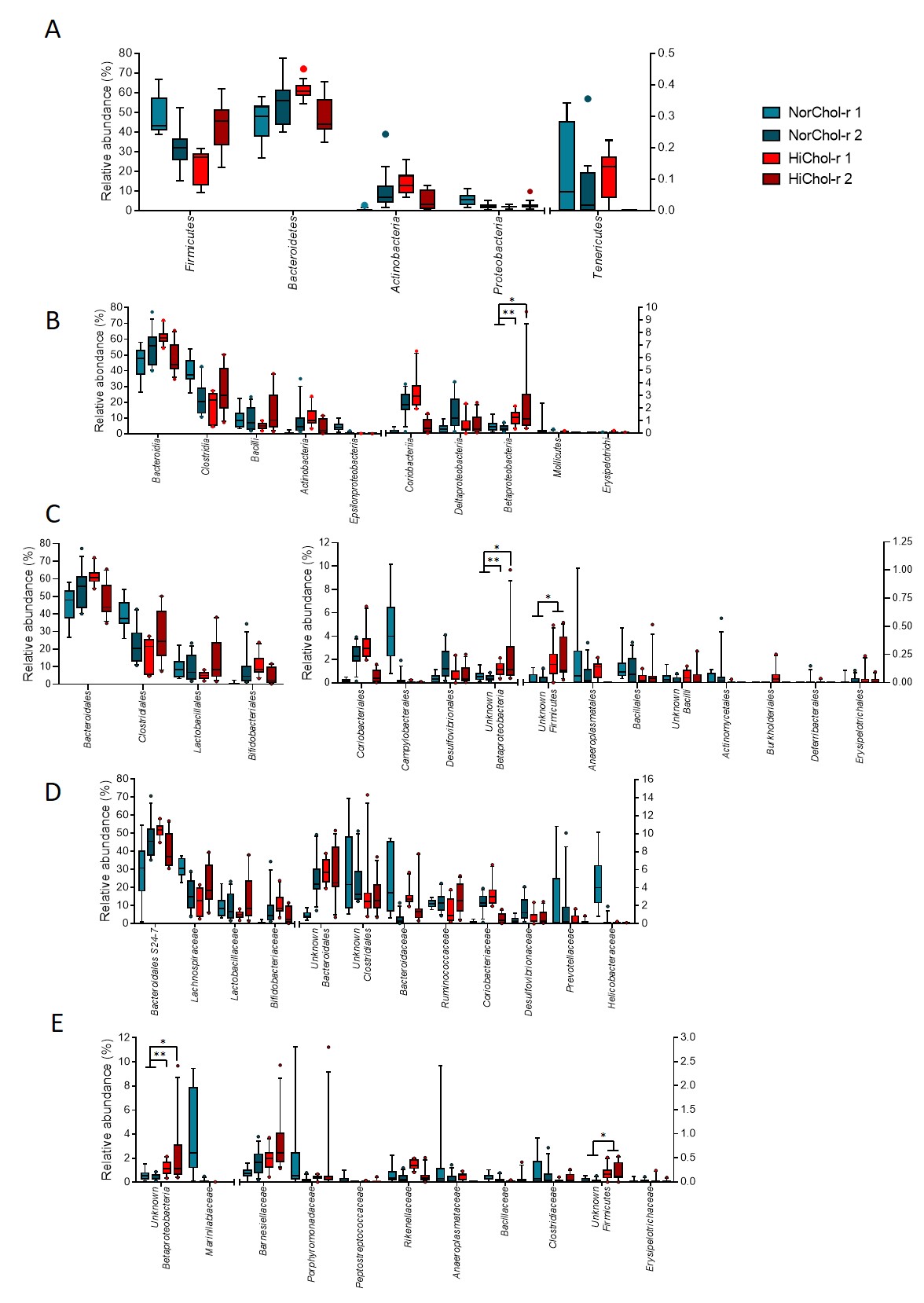

Supplement: Supplementary file 11 — Additional file 11: Figure S6. Fecal microbiota composition of normocholesterolemic and high cholesterol recipient mice. (A) Bacterial phyla distribution as percentage of total sequences in mice colonized with the microbiota from normo-cholesterolemic and high-cholesterol donors. (B) Bacterial classes distribution as percentage of total sequences in recipient mice (NorChol-r1 and r2, pictured cyan and dark cyan, HiChol-r1 and r2, pictured in red and dark red). (C) Bacteria orders as percentage of total sequences in recipient mice. (D) 12 most abundant families as percentage of total sequences in recipient mice. (E) 12 less abundant families as percentage of total sequences in recipient mice. (E) Data are represented as box and whiskers (10–90 percentile), n = 10–12 mice / group. * p < 0.05, ** p < 0.01, *** p < 0.001. [file 12915_2019_715_MOESM11_ESM.jpg]

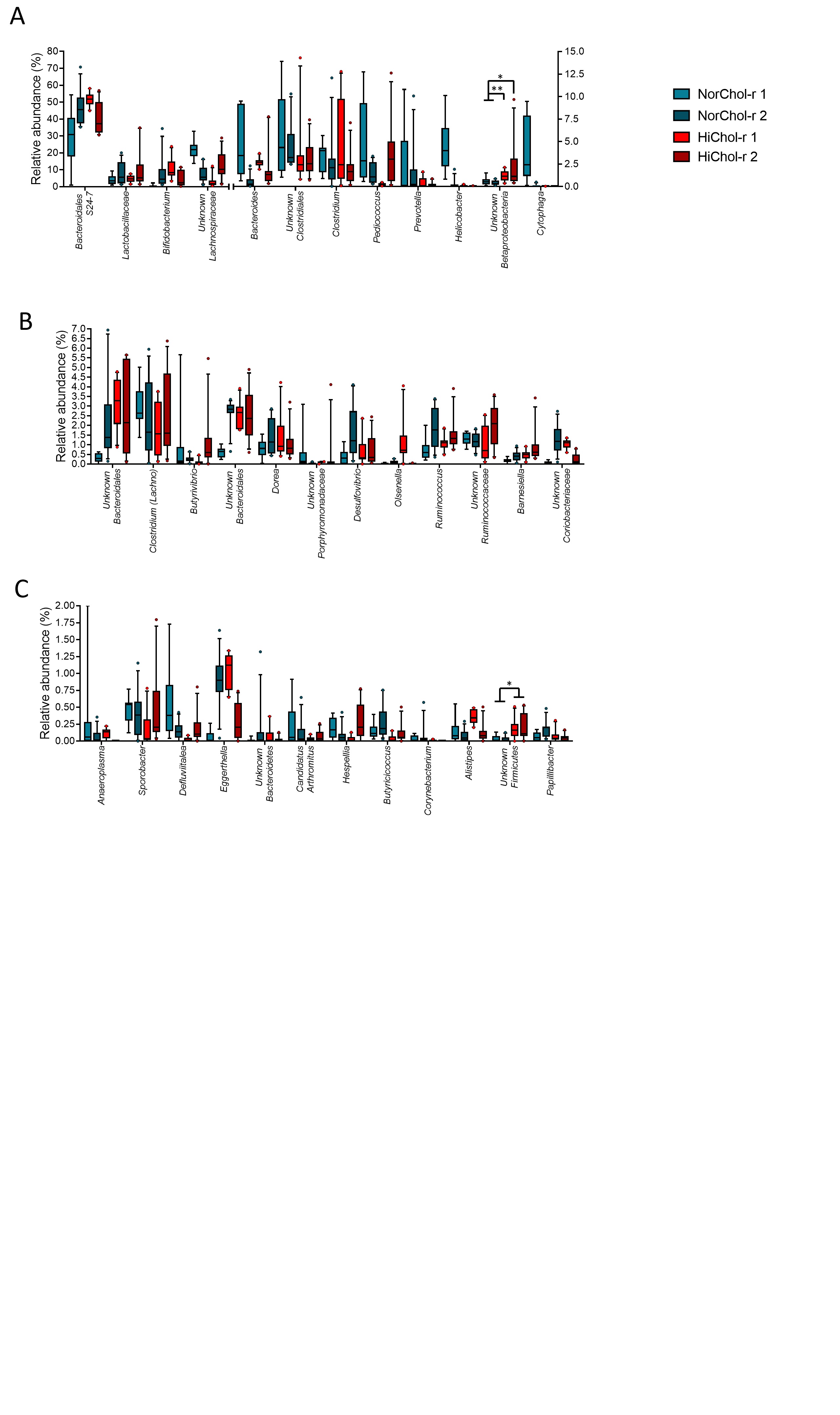

Supplement: Supplementary file 12 — Additional file 12: Figure S7. Fecal microbiota composition of normocholesterolemic and high cholesterol recipient mice. (A) 12 most abundant genera as percentage of total sequences in recipient mice (NorChol-r1 and r2, pictured cyan and dark cyan, HiChol-r1 and r2, pictured in red and dark red). (B) 12 less abundant genera as percentage of total sequences in recipient mice. (C) 12 low abundant genera as percentage of total sequences in recipient mice. Data are represented as box and whiskers (10–90 percentile), n = 10–12 mice / group. * p < 0.05, ** p < 0.01, *** p < 0.001. [file 12915_2019_715_MOESM12_ESM.jpg]
